# Modelling the Spread of Foot and Mouth Disease in Different Livestock Settings in Italy to Assess the Cost Effectiveness of Potential Control Strategies

**DOI:** 10.3390/ani15030386

**Published:** 2025-01-29

**Authors:** Michele Pesciaroli, Alessandro Bellato, Alessandra Scaburri, Annalisa Santi, Alessandro Mannelli, Silvia Bellini

**Affiliations:** 1 Istituto Zooprofilattico Sperimentale della Lombardia ed Emilia-Romagna, Via A. Bianchi 9, 25124 Brescia, Italy; michele.pesciaroli@izsler.it (M.P.); alessandra.scaburri@izsler.it (A.S.); annalisa.santi@izsler.it (A.S.); 2 Department of Veterinary Sciences, University of Turin, Largo P. Braccini 2, 10095 Grugliasco, Italy; alessandro.bellato@unito.it (A.B.); alessandro.mannelli@unito.it (A.M.)

**Keywords:** FMD, TAD, vaccination, livestock density, contingency plan

## Abstract

Foot and Mouth disease (FMD) transmission is strongly related to the distribution and density of FMD-susceptible animals. In this study, we applied a model to simulate the spread of FMD in three Italian regions with different livestock demographics (densely, medium, and sparsely populated) in order to assess the cost effectiveness of the disease control options within the framework of the Italian contingency plan. The results of the simulations showed that stamping-out was sufficient to control the outbreak in the sparsely and medium populated areas whereas, in densely populated areas, only vaccinations could effectively control an outbreak. We also estimated the economic resources needed for the adoption of different control strategies, considering livestock indemnity, cleaning, disinfection and disposal, surveillance, vaccination and personnel costs. The results obtained showed that indemnity due to culling had the highest impact on the costs in all scenarios and vaccination resulted in the most cost-effective option to apply in densely populated livestock areas. The results of this study can be a useful aid to Competent Authorities in the preparation of contingency plans against FMD.

## Simple Summary

The epidemics of Foot and Mouth disease (FMD) have dramatic environmental, social and economic consequences. The structure and density of FMD-susceptible species populations are crucial for disease spread and, in turn, for the choice of the control strategy to apply. The aim of this study was to compare the effectiveness and the cost of different FMD control options in containing an FMD outbreak in three areas of Italy with different livestock demographics. We found that a control option based only on the culling of animals becomes ineffective as the livestock density increases and only vaccination can curb disease spread. Furthermore, compensation for animal culling contributes the most to the cost of disease control. These results can serve as evidence for Competent Authorities to design a contingency plan and adopt the most cost-effective FMD control strategy.

## 1. Introduction

Foot and Mouth disease (FMD) is an infectious disease of cloven-hoofed species that is extremely contagious once introduced in a susceptible population. FMD is considered the protype of the Transboundary Animal Diseases (TADs), and the consequences of its incursion in FMD-free countries are so serious that the virus has even been considered a potential weapon of bioterrorism [1]. The disease has been eradicated from the territory of the European Union (EU) and its Member States are recognized as ‘Free from FMD without vaccination’ by the World Organization for Animal Health (WOAH). Nevertheless, FMD is still common in a large part of the world. The most recent outbreaks of FMD in the EU—which were rapidly controlled—were recorded in 2011 in a south-eastern region of Bulgaria close to the border of Türkiye [2]. 

The legal and illegal trade of live animals, as well as animal food products or by-products, pose a risk of introducing FMD to FMD-free countries. Therefore, in certain countries, risk assessments have been carried out to identify possible pathways of entry and quantify the associated risk [3,4,5]. Along with reducing the risk of entry, it is essential to develop contingency plans to promptly counteract any outbreak of FMD regardless of possible political, economic, and social pressures. To this end, several FMD models have been developed to simulate the spread of FMD in a susceptible population to compare the effectiveness of different control measures and to estimate the size and the cost of an outbreak [6,7,8,9].

Based upon current EU legislation, the measures adopted to control possible FMD outbreaks are based on the strategy of killing the infected herds and any animals in contact with them (stamping out), coupled with appropriate disposal or management of potentially infective animal products, such as food products, animal by-products, persons, vehicles, farm fomites, and any other substance liable to transmit the virus. In certain circumstances, this basic control strategy alone might not be sufficient to curb the disease and, therefore, if supported by risk assessment and sound economic considerations, additional actions can be implemented, such as the preventive culling of at-risk herds and emergency vaccination. However, since the FMD virus is highly infectious and the reaction in case of incursion must be swift, certain control activities should be implemented in peacetime. This is particularly relevant in Densely Populated Livestock Areas (DPLAs) including susceptible species, where the speed of intervention becomes crucial.

The hypothesis behind our study is that the impact of FMD incursion in Italy varies depending on the area of the primary outbreak. Italian livestock production is characterized by a heterogenous geographical distribution, with some of the most densely populated livestock areas in Europe found in Northern Italy (Po River valley), where cattle and pig farming are concentrated. On the other hand, certain areas predominantly in Central and Southern Italy are distinguished by herds of different species reared in extensive and traditional husbandry systems. 

In the framework of our research project, we applied a model to simulate the spread of FMD in three Italian regions with different livestock demographics (densely, medium, and sparsely populated livestock areas) to assess which control measure would best curb the epidemic in different epidemiological situations. Finally, the cost of control strategies was assessed with the aim of providing decision makers with the relevant information necessary to develop a contingency plan.

## 2. Materials and Methods

### 2.1. Source of Data

The geographical coordinates, animal species farmed, type of production, and number of cloven-hoofed livestock animals and farms in the areas included in the study were collected from the Italian National Database of Livestock Species [10] where the farms are classified as indicated in Table 1. Similar classification has been used in previous studies [9].

### 2.2. Between-Farm Infection Dynamics

The spread of FMD was simulated through an agent-based, compartmental SEIR (Susceptible-Exposed-Infected-Removed) model based on a dynamic network, as previously developed by Rossetti in 2018 [11]. The farm and the date were the epidemiological and time unit of interest. The geographical coordinates of the farms served to create an adjacency matrix on which a network was built with the following method. The network is made of nodes and arcs, the nodes represent the farms and the arcs represent the potentially infectious contacts between pairs of farms. Local transmission has a primary role in the spread of FMD. Therefore, in line with previous studies [12,13,14], we developed a model focused on this mode of transmission.

The probability of potentially infectious FMD contact between holdings accounts for a kernel of distance between farms, infectivity, and susceptibility of the animal species farmed and the herd size. 

The component of probability of infection due to distance, indicated as *w_ij_,* is modelled by means of a kernel function (1) and assumes values between 0 and 1:

(1) $$w_{ij}=\frac{k_{0}}{{1+\left(\frac{h_{ij}}{r_{0}}\right)}^{\alpha }}$$

where *h_ij_* is the distance between farm *i* and each of the other farms included in the model *j*; *k*_0_ is the value of *w_ij_* when the distance *h* equals 0; *r_0_* is the distance when *w_ij_* equals 0.50; *α* is the decay parameter that gives the shape to the kernel. The values of *k*_0_ = 1, *r*_0_ = 2500 metres and *α* = 2.3 were adapted from those previously reported [9,15] in order to obtain a smooth definition of local clusters as the distance from other farms increases. The value of *r*_0_ = 2500 metres was chosen in view of the subsequent reduction in transmission probability, following the inclusion of the effects of infectivity and susceptibility of infectious and susceptible herds, respectively (see Section 2.2.1). 

#### 2.2.1. Susceptibility and Infectivity of Farms According to Their Attributes

The effect of certain attributes of the farm, which are relevant to the spread of the infection—such as animal species, type of production, size of the herd—on the probability of infection p(a) and the associated uncertainty are modelled by means of a Beta distribution (Table 2). 

The values defining the Beta distributions were retrieved from scientific publications [9,16]. The effect of vaccination—calculated based on studies estimating vaccine efficacy and associated uncertainty [16,17,18,19,20]—was included by reducing farm susceptibility and infectivity by 40%.

#### 2.2.2. Dynamic Network

Once the probability p(*a*) of infection was obtained, a random number between 0 and 1 was extracted; if its value was above the value of p(*a*), the arc was created and the infection can be transmitted.

For each simulation run, the probability of transmission was recalculated and, consequently, a new network was created, resulting in a dynamic network that changed over time. The dynamic network was created by running 100 simulations. 

#### 2.2.3. SEIR Model

Each of the networks of infectious contacts was used to run a SEIR model in which the farms are classified according to the following statuses: Susceptible, Exposed, Infected, Removed.

The parameters governing the transition between statuses are summarized in Table 3. 

The latency period, driving the transition between the statuses Exposed and Infected, was set at 2 days. Ten days were considered the time necessary to cull and dispose of animals [16,21].

The model was run for 60 days; for each day, we calculated the median of infected farms, the first and third quartiles, and the 95th percentile.

### 2.3. Areas Included in the Study

Three areas were selected to explore the dynamics of the epidemics and the effect of the control strategies. The areas differ in terms of livestock population density, species, and type of farming. With regards of the density of cloven-hoofed species, the areas were classified as the following:

1. Densely Populated Livestock Area (DPLA), which includes the administrative province of Brescia, Mantua, and Cremona in the region of Lombardy; > 450 animals/km^2^;
2. Medium Populated Livestock Area (MPLA), which includes the administrative province of Pavia and Lodi in the region of Lombardy; >51 animals/km^2^;
3. Sparsely Populated Livestock Area (SPLA), which includes the administrative provinces of Grosseto and Siena in the region of Tuscany; ≤ 50 animals/km^2^.

The areas were classified by slightly altering the cut-off previously indicated by Huirne and Horst [22] and decreasing the lower limit of MPLA from 150 animals/km^2^ to 51 animals/km^2^.

The parameters of the livestock demography of the three areas are reported in Table 4.

### 2.4. Strategies of FMD Control and Model Simulations

The simulation of the model started with the index case located in the most densely populated of the three areas. The time required to detect the primary outbreak of FMD has been described as variable [17,18,23]; in this study, it was set at 10 days, which is the mean value of those reported. At day 20 of the simulation, at the end of the detection time and infectious period, the status of the farms was changed to *Removed*. 

In accordance with current EU legislation (Regulation on transmissible animal diseases, Regulation (EU) 2016/429 [24], rules for the prevention and control of certain listed diseases, Commission Delegated Regulation (EU) 2020/687) [25] and the control strategies tested were as follows:

1. Stamping-out (SO)
2. Pre-emptive culling (PC) of 50 farms at risk of being contaminated or likely to contribute to the spread of the infection within a radius of 5 km;
3. Ring vaccination (V) within a radius of 5 km. Vaccine efficacy was set at 40%.

The start of the model where PC is applied was set at 50 farms infected and 50 removed in order to consider the time required for culling, which we estimated to take 10 days. In the case of a V scenario applied to the DPLA, the simulations began with 100 infected farms, which takes into consideration the time required to vaccinate and for a protective immune response to be established, which we set at 17 days.

The effect of the control strategy on the epidemics was assessed at 30 days of simulations.

### 2.5. Direct Costs of FMD Control and Model Simulations

The direct costs of the FMD control options applied were estimated using a deterministic approach and adapting a spreadsheet tool developed by Casal (2022) [26], where the total costs were the result of (1) animal compensation due to culling, (2) cost of culling, carcass disposal, farm cleaning, and disinfection, (3) cost of surveillance, (4) cost of vaccination (when applied), and (5) cost of personnel. The costs of the operations performed on farms were estimated using the median number of farms culled for each type under the different scenarios after 100 simulations. All the farms not removed were visited once for surveillance purposes. In the case of V, all the farms not infected or removed were considered eligible for vaccination. Compensation due to culling was obtained by multiplying the median number of farms subject to culling by the median of the animal population for each farm type. The prices of each animal category, the time, human and materials resources required to carry out the operations foreseen are given in the Supplementary Materials (Tables S1–S5). The costs of the epidemics under the three control scenarios were assessed at 60 days of model simulations. 

### 2.6. Software

The SEIR model, based upon a dynamic network, was implemented using the Ndlib library of Python programming language [27]. The different scenarios were set in R (version 4.4.2) [28] before starting the simulations with Python.

## 3. Results

In the SPLA and MPLA, the control strategy based on SO curbed the disease within 30 days. Whereas, in the DPLA, SO was not sufficient enough to halt the transmission, hence all the control strategies were tested. The results of the DPLA allowed us to carry out a cost-effectiveness comparison of the possible disease control options.

### 3.1. SPLA

**Figure 1:**
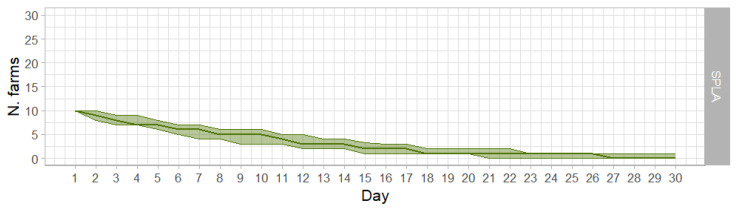
Epidemic curves of the SPLA; median values (solid line) and interquartile range values (shaded areas) of the infected farms per day in the SPLA applying SO.

FMD was only transmitted if the simulation started with 10 infected holdings; with fewer numbers of infected farms, no infectious arcs are created and transmission does not take place because of this. At day 30, there was one infected farm only in the 3rd quartile of the simulations. The median and 1st quartile reached 0 at day 27 and days 21 (Figure 1).

### 3.2. MPLA

**Figure 2:**
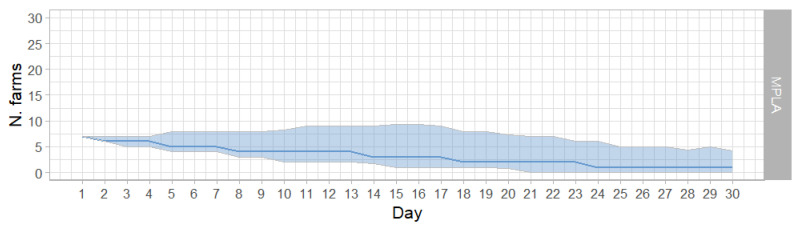
Epidemic curves of the MPLA; median values (solid line) and interquartile range values (shaded areas) of the infected farms per day in the MPLA applying SO.

The simulation started with seven infected farms; for lower numbers of outbreaks, the epidemic does not occur. The number of infected farms declined over the simulation period. At day 30, the median, the first, and third quartile were 1, 0, and 4, respectively (Figure 2).

### 3.3. DPLA

#### 3.3.1. DPLA and Stamping-Out (SO)

**Figure 3:**
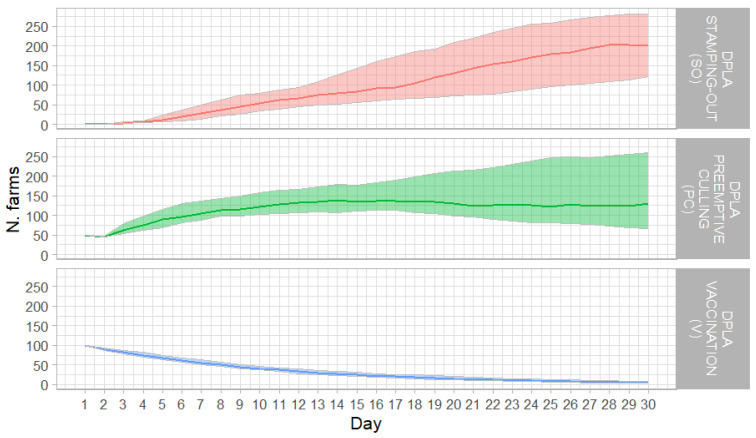
Epidemic curves for the three control strategies; median values (solid line) and interquartile range values (shaded areas) of the infected farms per day in the DPLA applying SO (upper panel), PC (central panel), and V (lower panel).

When FMDV was introduced in a DPLA, the number of infected farms increased rapidly. At day 10 of the simulation, when SO was applied to control the epidemic, the median, 1st, and 3rd quartile of infected farms were 29, 14, and 49, respectively (Figure 3). 

Afterwards, the spread of FMD slowed, though the number of infected farms continued to rise up to day 30, when the median, the 1st, and 3rd quartile were 200, 122, and 182, respectively.

#### 3.3.2. DPLA and Preventive Culling (PC)

To simulate this control option, the model was set with 50 infected herds and 50 culled for preventive purposes (Figure 1). The simulation started at 10 days after the first FMD event, i.e., 20 days after the introduction of FMD. At two weeks from the beginning of the simulation, the median, 1st, and 3rd quartile of infected farms were 138, 107, and 179, respectively. Afterwards, the number of infected farms declined only in the 1st quartile of the simulation; otherwise, the numbers remained substantially constant until day 30.

#### 3.3.3. DPLA and Vaccination (V)

In this control scenario, the simulation started at day 17, after the diagnosis of the primary outbreak when 100 holdings are already infected, which occurs 27 days after the introduction of FMD. The median, 1st, and 3rd quartile of the number of infected herds steadily declined over the simulation period that ended with 6, 4, and 8 farms, respectively (Figure 3).

### 3.4. Direct Costs of FMD Control in the DPLA 

**Figure 4:**
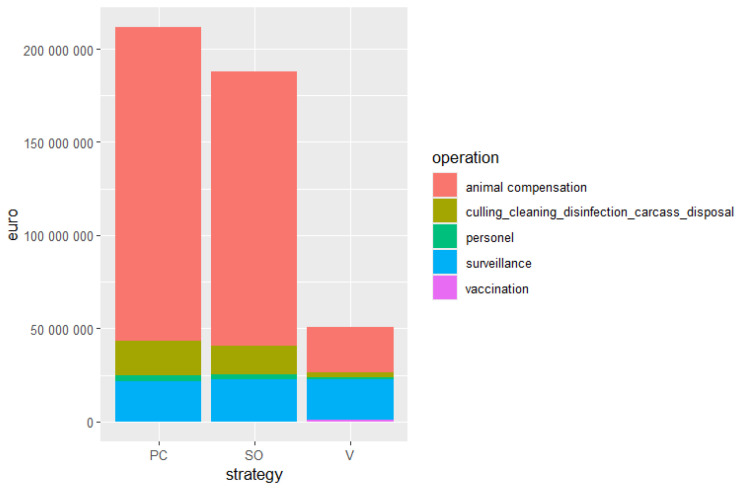
Cost of stamping-out (SO), stamping-out and preventive culling, and (PC), stamping-out and vaccination (V) applied to control the disease in the DPLA.

At the end of the simulation period 571, 689, and 99 farms are subject to culling under the SO, PC, and V scenario, respectively. Compensation due to culling had the highest impact on the cost of all the scenarios considered (Figure 4). 

Surveillance is the second most expensive activity, with comparable costs across all scenarios: C (€ 22,602,080), PC (€ 21,851,080), and V (€ 21,841,280).

The costs of cleaning and disinfection and carcass disposal are proportional to the number of culled farms with an expenditure of € 15,217,224 (C), € 18,385,198 (PC), and € 2,640,004 (V). These activities do not appear to be a financial constraint, accounting for 8.1%, 8.7%, and 5.2% of the total cost of each intervention strategy in the case of SO, PC, and V, respectively.

## 4. Discussion

In this study, we investigated the impact of the introduction of FMD in three areas of Italy with different livestock demographics. The results of the simulations showed that population composition and, most importantly, density influence the evolution into an epidemic and determine the failure or success of the control strategy applied, whereas this control option combined with preventive culling did not control FMD in a DPLA, where only vaccination successfully contained the spread of the infection.

The population density of susceptible animals plays a crucial role in determining the evolution of the infection in an epidemic and this is particularly true for TADs such as avian influenza [29] or FMD [29,30]. The close relationship between distance between farms and probability of infection guided the decision to adopt a disease spread model based on local transmission of the infection, given that one of the objectives of the study was also to determine the population density threshold of FMD-susceptible animals for implementing emergency vaccination. As stated in the introduction of the manuscript, for the purposes of the study, animal movements were not included in the model. Indeed, we adopted an approach similar to the one developed by the European Food Safety Authority (EFSA) to assess the effectiveness of control measures of the category A diseases in the Animal Health Law, including FMD [14] and in previous studies in the UK and Japan [13]. We are aware that this pathway of transmission would have a relevant impact in MPLA and SPLA where, because of the lower density, local transmission is less efficient. Not surprisingly, simulations of disease spread in those areas have to start with a few infected farms. A further refinement of the study would include incorporating a sensitivity analysis of the effect of time of FMD detection.

Many studies have compared the strategies to control FMD outbreaks in FMD-free countries [23,31,32,33] and evidence suggests that a single solution to achieve eradication does not exist. Indeed, the selection of the most appropriate control strategy time of intervention must take into account livestock demographics, evolution of epidemics, resource availability, and the legal framework that regulates the control options. Previous studies have found that as the animal population density increases, ring culling or pre-emptive culling based on risk has proven to be effective [18,33]. However, the required radius of the ring area and the required threshold density are not absolute and undisputable but need to be adjusted according to the epidemic dynamics in order to avoid over-culling [31]. In our study, the pre-emptive culling of 50 farms within a radius of 5 km from the index case did not stop the epidemic in the DPLA and resulted in higher numbers of culled animals. We decided not to assess the impact of removing more than 50 farms since it would have increased the number of culled animals and the cost of the control strategy. In fact, culling demands significant human resources and carcass disposal capacity and can cause a bottleneck that hinders the completion of this control strategy. This event occurred in the Netherlands during the epidemic of 2001, when ring culling was replaced by vaccination as soon as it was realized that carcass destruction capacity was insufficient to deal with the epidemic [34]. In addition, whenever SO or preventive depopulation measures are applied to contain FMD, it has to be taken into account that the mass culling of animals leads to harsh public debate, given the divergent views in society on the value of animal rights, leaving policy makers with the hard task of deciding on the best disease control measures combining social acceptance with science, ethics, and economics [35]. 

In DPLAs, emergency vaccination was found to be the only strategy able to control the disease and is associated with the lower direct costs. From the results obtained, it emerged that vaccination controlled the spread of the infection even when a cautious efficacy of vaccination was applied (40%), which is extremely conservative compared to the assumptions in previous studies [9,18,23]. 

However, this control measure would come up against negative opinion against eating meat from vaccinated animals. Data from a Eurobarometer [36] report showed that a large portion of consumers would hesitate to consume meat from vaccinated animals. Indeed, more than 50% of the responders believed that the consumption of meat from vaccinated animals is related to some health risk. The aim of this study was to support central veterinary authorities in the preparation of contingency plans and, given the FMD-free status of Italy, the study focused on the early phase of the infection. Accordingly, the model simulation and analysis of the cost-effectiveness of the control strategy did not consider the evolution of disease into an endemic situation.

## 5. Conclusions

Maintaining an entirely non-immune population of animals susceptible to FMD requires permanent disease awareness and preparedness. Modelling disease spread lays the foundations for the design of a sound contingency plans and for the rapid mobilization of resources to respond to an outbreak. Contingency plans have proved to be a crucial tool for the successful control of disease emergencies and, considering that a solution for all the possible situations does not exist, such plans must be flexible enough to adjust to the circumstances.

Past animal health crises have shown the benefits of having specific, detailed, and rapid procedures for the management of disease emergencies and it is well documented that FMD-free countries capable of responding promptly to an FMD incursion recover their free status faster than countries with longer reaction times, as highlighted in a study by WOAH [37].

## Figures and Tables

**Table 1:** Farm categorization used in the study.

Farm	Number of Animals
Large dairy bovine	>31
Large cattle	>31
Small size bovine farms	≤30
Water buffalo	≥11
Small ruminants	≥11
Large swine fattening	>31
Large swine breeders	>31
Small swine	≥11 and ≤30
Backyard swine	≤11

**Table 2:** Susceptibility and infectivity parameters of the farms type.

Farm	Susceptibility	Infectivity
Large dairy bovine	Beta (90, 10)	Beta (45, 55)
Large cattle	Beta (90, 10)	Beta (45, 55)
Small size bovine	Beta (90, 10)	Beta (25, 75)
Water buffalo	Beta (90, 10)	Beta (45, 55)
Small ruminants	Beta (15, 85)	Beta (25, 75)
Large swine fattening	Beta (6, 94)	Beta (90, 10)
Large swine breeders	Beta (6, 94)	Beta (90, 10)
Small swine	Beta (6, 94)	Beta (45, 55)
Backyard	Beta (5, 95)	Beta (5, 95)

**Table 3:** Transmission parameters used in the SEIR model.

Model Parameter	Parameter	Value
Transmission Rate between Infectious to Susceptible	β	0.25
Transition rate between Exposed to Infected	1/α	0.5
Transition Rate between Infected to Removed or Removal Rate of Infectious	1/γ	0.1

**Table 4:** Livestock demographics of the three study areas. Median, minimum, and maximum numbers of animals for each farm type are reported.

Measurement	DPLA	MPLA	SPLA
Large dairy bovine farms	1521	801	49
Large cattle farms	696	167	52
Small size bovine farms	408	474	268
Water buffalo farms	5	8	3
Small ruminants farms	88	284	667
Large swine fattening farms	651	192	3
Large swine breeders farms	223	88	17
Small swine farms	44	76	53
Backyard swine farms	6	32	529
Total farms	3642	2122	1641
Surface (km^2^)	4.024	7.477	4.823
N. Farms/km^2^	0.91	0.28	0.34
Large dairy bovine	51–211–2096	51–204–2130	51–122–579
Large cattle	51–225–3710	51–92–3112	51–86–432
Small size bovine	11–27–50	11–22–50	5–19–50
Water buffalo	50–107–239	18–230–828	119–177–457
Small ruminants	11–39–2407	11–24–1912	11–158–5729
Large swine fattening	53–1841–23,608	51–1290–19,100	312–1300–3629
Large swine breeders	51–2318–17,998	62–1391–17,735	54–94–443
Small swine	11–18–50	11–17–50	5–11–48
Backyard swine	11–12–12	11–12–19	1–1–14
Total animals	3,038,809	875,230	197,239
Surface (km^2^)	4024	7477	4823
N. Animal/km^2^	755	117	40

## Data Availability

All data are available within the manuscript or provided as Supplementary Materials.

## Supplementary Materials

The following supporting information can be downloaded at: https://www.mdpi.com/article/10.3390/ani15030386/s1, Table S1. Animal compensation. Median number of culled farms in the DPLA after 60 days of simulation applying SO, PC, and V control. Table S2. Cost of animal compensation, culling, cleaning, and disinfection operations and carcass disposal (€). Table S3. Cost of surveillance and vaccination operations (farm) (€). Table S4. Composition of personnel for specific operations. Table S5. Working days to complete the operations (days) [38].
