# Supplementary material for: Modelling the Spread of Foot and Mouth Disease in Different Livestock Settings in Italy to Assess the Cost Effectiveness of Potential Control Strategies
Source: Animals (Basel). 2025 Jan 29;15(3):386. doi: 10.3390/ani15030386 (PMC11816357; doi:10.3390/ani15030386)
Supplement: Supplementary file 1 [file animals-15-00386-s001.zip › animals-3403029-supplementary.pdf]

animals

MDPI

Supplementary Materials:

Table S1. Animal compensation. Median number of culled farms in the DPLA after 60 days of simulation applying SO,PC and V control.

Culled Farms (median)	Stamping-out (SO)	Pre-emptive Culling (PC)	Vaccination (V)
Large dairy bovine farms	311	376	54
Large cattle farms	144	174	25
Small size bovine farms	81	97	14
Water buffalo farms	0	0	0
Small ruminants farms	6	7	1
Large swine fattening farms	23	28	4
Large swine breeders farms	6	7	1
Small swine farms	0	0	0

Table S2. Cost of animal compensation, culling, cleaning and disinfection operations and carcass disposal (€).

	Compensation (animal)[1]	Culling, Cleaning and Disinfection (farm)[2]	Carcass Disposal (animal)[2]
Large dairy bovine farms	1150	3,500	120
Large cattle farms	1625	3,500	130
Small size bovine farms	1388	1,200	120
Water buffalo farms	-	-	-
Small ruminants farms	183	1,200	12
Large swine fattening farms	230	1,500	20
Large swine breeders farms	325	1,500	20
Small swine farms	-	-	-

Table S3. Cost of surveillance and vaccination operations (farm) (€).

	Surveillance (farm)[2]	Single Vaccine Dose (animal)[2]
Large dairy bovine farms	6,600	2.5
Large cattle farms	6,700	2.5
Small size bovine farms	3,200	2.5
Water buffalo farms	3,200	2.5
Small ruminants farms	4,000	2.5
Large swine fattening farms	10,900	2.5
Large swine breeders farms	11,300	2.5
Small swine farms	5,100	2.5

Table S4. Composition of personnel for specific operations.

	**Team Composition**		
**Activity**			
	Veterinarian	Technician	Other Profession
Coordination	2		
Surveillance	2		
Infected premises depopulation	2	1	1
Vaccination	2		

Table S5. Working days to complete the operations (days).

	**Culling, Cleaning and Disinfection (farm)[2]**	**Surveillance (farm)[2]**	**Vaccination (farm)[2]**
Large dairy bovine farms	7	2	0.5
Large cattle farms	6	2	0.5
Small size bovine farms	1.8	2	0.3
Water buffalo farms	3.5	2	0.5
Small ruminants farms	5	2	0.4
Large swine fattening farms	10	2	0.5
Large swine breeders farms	10	2	0.5
Small swine farms	1	2	0.2

[1]Animal compensation prices are those paid by the Italian animal health authorities for culling [38]; [2] Estimate based on those previously reported [34] and also assessed by the Italian animal health authorities.
